# Effects of Online Fan Community Interactions on Well-Being and Sense of Virtual Community

**DOI:** 10.3390/bs13110897

**Published:** 2023-10-30

**Authors:** Min Sung Kim, Soyoung Wang, Seongcheol Kim

**Affiliations:** 1 School of Media and Communication, Korea University, Seoul 02841, Republic of Korea; okminsung@naver.com; 2 Service Biz Group, Digital Appliances, Samsung Electronics, Suwon 16677, Republic of Korea; soyoung1007@naver.com

**Keywords:** fan community platform, mental well-being, sense of virtual community, fan interaction, parasocial relationship, Weverse

## Abstract

Activities in the digital economy driven by information technology have rapidly increased in scope and speed in the aftermath of COVID-19. Meanwhile, social isolation accelerated by quarantine measures has increased concerns about individuals’ mental health. However, little is known about the specific consequences of online interactions, especially when applied in online fan community-based relationships. Therefore, we examined the impact of loneliness in the context of COVID-19 on online interaction with other fans and parasocial relationships with celebrities on the Weverse platform. We also examined how these interactions influence mental well-being and the sense of virtual community. With 202 valid data samples acquired from global BTS fandom, this study conducted a partial least squares–structural equation modeling analysis. The empirical results demonstrate a significant positive relationship between loneliness and the extent of online interaction, while no significant impact on parasocial relationships was observed. Both online interaction and parasocial relationships were found to enhance both well-being and SOVC. However, these results were observed to differ between Weverse paid subscribers and free users.

## 1. Introduction

With the outbreak of COVID-19, governments in many countries have adopted various types of physical distancing measures that restrict the outdoor activities of citizens. The pandemic has accelerated non-face-to-face digital transformation in all fields, including shopping, education, work, and leisure [1,2]. These changes have led to increased individual isolation, and individuals’ subjective feelings about social isolation during COVID-19 have been found to have a close relationship with adverse psychological reactions [1,3]. A high level of loneliness has the potential to trigger psychological disorders such as depression, stress, and anxiety [4,5,6], ultimately negatively impacting an individual’s overall well-being [7,8].

Accordingly, many government officials and scholars have encouraged people to use online spaces to cope with mental health problems. For instance, the United Nations provided guidelines to protect individuals’ mental health and promote a sense of belonging by participating in discussions within online communities [9]. The previous literature has argued that social interactions in emerging media services can significantly enhance people’s sense of belonging and connection, especially under conditions of restricted face-to-face contact [10,11].

However, there have been contradictory findings on the influence of online interaction. Some scholars have explained that the risk of interacting with misinformation in a virtual space [12] or the passive use of social networking services (SNSs) [13] could negatively affect an individual’s well-being. There is also a lack of empirical research applicable to the current pandemic situation, and that sheds light on the results of online interactions in specific contexts. For example, prior studies have discovered the influence of the use of emails and phones [11], SNSs [13], or massive multiplayer online role-playing (MMORPG) games [14] on individuals’ well-being or belongingness during COVID-19. Nevertheless, few studies have investigated the influence of diverse online interactions within celebrity fan communities on the mental well-being of fans.

Online fan communities in the South Korean entertainment industry have experienced several noticeable changes over the last few years. The fandom business model has primarily moved to grow platforms and provide content. Specifically, beyond solely presenting offline experiences, like concerts or TV music programs, to the fans of celebrities, entertainment companies have carefully devised ways to promote K-pop fans’ engagement in a virtual world.

Subsequently, whereas previous online fan activities were sporadic on various social media platforms, the new version of the fan community platform combined all of the online fan activity functions. For instance, it allows interactive communications between K-pop fans and celebrities, fast access to the management agencies’ official information, and easy purchase of celebrity-related products. The emergence of such a space allows fans to move to new types of fandom platforms with diverse value offerings. In fact, given the effort of management agencies and platform operatives to promote discussions on the fandom platform, most fan–celebrity and fan–fan interactions have begun to frequently take place on representative fan community platforms, such as Weverse, LYSN, and Universe [15]. Extant fan studies have demonstrated the association between belonging to online fan communities and fans’ positive mental states [16,17]. However, they have not incorporated the characteristics of newly emerging fan community platforms and have not considered the changes in individuals’ psychological states brought about by the COVID-19 pandemic. Therefore, the purpose of this study is to address these gaps and examine how diverse online interactions in fan community platforms impact two crucial components of community psychology: individual well-being and the sense of virtual community.

Specifically, we aim to explore the influence of online interactions within fan community platforms, taking into account the personal loneliness experienced during the pandemic. We further delineate these online interactions within newly emerging fan community platforms as interactions with other fans and parasocial interactions with celebrities.

This study distinguishes itself by focusing on fan community platforms, which are at the forefront of the ever-evolving fandom business within the media industry. This growing significance of fandom not only influences the media sector but also has a profound impact on individuals’ lives. The evolving field of community psychology has the potential to play a significant role in community research by crafting efficient strategies for addressing some of the prevalent social challenges currently experienced or anticipated by communities in the future [18]. Considering the two vital factors in community psychology, well-being and the sense of virtual community, we anticipate contributing to the design of fan community platforms that can enhance individuals’ quality of life.

The rest of the paper is organized as follows. In Section 2, we delve into the characteristics of newly emerging fan community platforms and examine interactions with other fans and celebrities within these platforms. Additionally, we explore the concepts of well-being and the sense of virtual community. Section 3 outlines the research methodology employed in this study, while Section 4 presents the study’s findings. Section 5 is dedicated to the discussion and conclusions.

## 2. Literature Review

### 2.1. Fan Community Platform

A fan community platform has the characteristics of both online and fan communities. Previous studies have described online communities in various ways: Bagozzi and Dholakia [19] define them as virtual social spaces where members share their everyday purpose and create content, while Balasubramanian and Mahajan [20] highlight their spatial and temporal constraints. Online communities are often formulated based on regional societies, demographic characteristics, and various offline relationships but are also formed around shared interests, tastes, and preferences [21,22]. Online communities can be constructed based on individuals’ transactional relationships, interests, or fantasies [21]. For instance, people with a common preference for music and singers may assemble in an online fan community as a taste-based online community. 

An online fan community is a transcultural community where people from various countries, ethnicities, and cultural backgrounds share their tastes [23]. The development of such communities is driven by information technologies (IT) through various forms of content, such as text, photos, GIFs, and videos, shared and transmitted to millions of fans, creating an environment that maximizes online interaction [24]. Social media platforms, such as Twitter, have allowed fans to engage in personal communication more quickly and deeply [25]. Among all, K-pop singers’ powerful and global fandom actively employ every media channel to interact with other fans. A study of K-pop fandom communities reveals the role of social media platforms as strategic outposts for K-pop fans to promote their favorite artist’s songs or important messages with creative content [26]. Table 1 provides an example and explanation of the social media usage of some K-pop celebrity fans.

With more advances in IT, the fan community platform emerged in South Korea through leading management companies or game providers, with its fandom range expanded from local to international. Weverse, Bubble, and Universe appear to be the largest and most active platforms for fan activities, which have distinct and noteworthy attributes. For instance, since all these celebrity communities are officially operated by management agencies, official album-launching or concert ticket-selling information is easily shared with broader coverage. Also, the barrier to joining the fan community platform is comparatively lower than earlier fan cafes because a membership fee is not required. The fandom platform membership usually functions as a subscription for receiving premium celebrity-related content or merchandise. Lastly, these apps and websites allow easier and more complimentary access to the conversation between global fans and artists than the existing portal sites-based fan communities or SNSs [27]. In summary, a fan community platform is both an online community and a transcendent fan community established based on their interests and preferences for celebrities. It is a convergence of platforms, content, commerce, and new media technologies on which global fans focus. More descriptions and comparisons of each brand-new fan community platform are shown in Table 2.

### 2.2. Interaction with Other Fans and Celebrities

#### 2.2.1. Online Interaction with Other Fans

Fans’ online interactions can be understood as the behavior surrounding their mutual influence, sharing, supporting, and creating value within the online community [28]. With the advent of a new fan community platform like Weverse, fans’ interactions have become even more accessible, with a deeper process of exchanging influence. In particular, fan communication channels with a friendly and pleasant atmosphere have allowed them to freely express their opinions and emotions, activating more frequent interactions between community members [27]. For example, their frequency in uploading posts and leaving likes and comments on fellow fans’ posts can imply fandoms’ substantial involvement in the fan community platform. The content of the posts, which ranges from informative to daily life sharing and playful parodies, jokes, or memes, is likely to show the diversity and quality of fans’ communication [29]. Moreover, community members’ enthusiastic engagement is another noteworthy sign of the intensity of online interactions. One fandom platform, Weverse, provides an automatic translation function, supported in ten languages, helping global timely communication among global fans. In addition, BTS fans’ official lightstick, the “Adorable Representative MC for Youth” (ARMY) Bomb, could have been connected to Weverse when watching online concert videos, extending fans’ experience to other steps. Accordingly, the current study approaches online interaction as the intensity of engagement with the digital fandom community, considering the frequency and the quality of interaction with other fans.

#### 2.2.2. Parasocial Interaction with Celebrities

The concept of parasocial interaction speaks to the spectator’s, audience’s, or user’s feelings about interacting with a performer on the screen [30,31,32,33]. Within the context of the fan community platform, it refers to the process of interaction between fans and celebrities, often considered an essential part of the fan groups’ enjoyment [26]. Meanwhile, a parasocial relationship is formed by repeated parasocial interaction, meaning that fans’ feelings of intimacy are enhanced through repeated exposure to celebrities, eventually forming a non-existent intimate relationship [31].

Fans can interact directly or indirectly with celebrities on digital fan community platforms, leading to stronger parasocial relationships. These platforms support various communication functions that can connect fans and celebrities and build a robust relationship between the two. Specifically, fans can indirectly communicate with celebrities by leaving comments or likes on the posts or messages that celebrities leave on the platform. Fans can also send direct messages to a specific member in a group by adding hashtags like #to_BTSRapmoster below the post or through a one-on-one artist and fan conversation service in separate fan chat windows. The development of information and communication technology (ICT) even allowed real-time video-call fan engagement within the platform.

Various types of parasocial relationships have been studied by previous researchers [34,35], such as companionship, identification, friendship, understanding, and problem-solving [36]. In light of fans’ persistent desire to build relationships with celebrities on the fan community platform, this study specifically delves into the concepts of understanding and friendship, the most traditional types of parasocial relationships [31]. Fans may quickly learn about the artists through various communication features implemented in the service, subsequently enhancing their understanding of celebrities. Also, repeated celebrity interactions may lead to a sense of friendship, a mutual relationship based on intimacy and fondness [37]. Repeated, direct, and private exposure to celebrities may allow fans to feel a high level of favorability and psychological attachment toward these celebrities [36], thus deeming their relationship as friendships on the fan community platform [38].

#### 2.2.3. Loneliness and Interaction

Loneliness has become a prevalent emotional response, primarily as a consequence of the COVID-19 preventive measures [39]. The pandemic and quarantine measures imposed by the modern age have substantially transformed various aspects of our daily lives, including the way we engage in work and social activities [40]. The implementation of self-isolation, social distancing, and “stay-at-home” orders due to the pandemic has contributed to heightened feelings of loneliness for many individuals [41,42]. This emotional state of loneliness is defined as the unpleasant feeling that arises from a perceived mismatch between one’s actual and desired social connections [43].

Historically, individuals with limited social interactions or a greater sense of loneliness have turned to mass media to compensate for their social isolation [44]. These lonely individuals often use mass media as a means to fulfill their interpersonal needs [35,45]. This habit of communicating with media characters as if it were a two-way interaction is termed parasocial interaction [31].

Interestingly, this situation has become even more pronounced in the context of the COVID-19 pandemic. Empirical evidence reveals that, despite their increased isolation during the COVID-19 outbreak, individuals seem to have discovered various methods to mitigate their solitude, such as leveraging communication technology [42]. When face-to-face communication was unavailable, online interaction facilitated connections and mitigated psychological distress and feelings of isolation [46]. The widespread availability of smartphones with internet access has proven to be a valuable tool for obtaining information about the pandemic and maintaining social connections during quarantine [47]. Furthermore, the rise of online communities has significantly expanded the potential for distant individuals to communicate with one another [40]. This has enabled lonely individuals to actively seek and prefer online social interactions [48]. Therefore, this study posits the following hypotheses:

**H1.**  *Loneliness will be positively related to the level of online interaction within the fan community platform.*

**H2.**  *Loneliness will be positively related to the development of parasocial relationships on the fan community platform.*

### 2.3. Community Psychology and Well-Being

Community psychology has sought to shift the focus from studying individual psychology to understanding the individual within the context of a community [49,50]. Community psychology takes an ecological perspective to tackle social inequalities, with a specific emphasis on the fundamental power dynamics inherent in human interactions [51]. The fan–artist relationship has been marked by an inherent imbalance and a distinct division between artists and fans, underscoring an aspect of inequality from an ecological perspective. Artists typically had no say in choosing their fans, could not easily terminate such relationships, and mutual admiration was not a common feature. Therefore, the conventional artist–fan relationship did not exhibit characteristics like symmetrical reciprocity, communion, or solidarity [15,52]. However, recent fan community platforms have been instrumental in bringing about changes in these aspects, transforming the nature of the fan–artist relationship.

Community psychology aims to employ insight to enhance the overall well-being of individuals [53,54]. In the context of the COVID-19 pandemic, the importance of community interactions for well-being becomes particularly evident. Interactions within communities, whether taking place in local neighborhoods or in virtual spaces, can be regarded as constructive strategies aimed at fostering the well-being of both individuals and communities amidst the challenges posed by the pandemic [46]. Well-being, a key theme in community psychology [55], is defined as “a positive state of affairs, brought about by the simultaneous and balanced satisfaction of diverse objective and subjective needs of individuals, relationships, organizations, and communities” [56] (p. 2). From the viewpoint of positive psychology, well-being contributes significantly to maintaining an optimal state among individuals and organizations, including hedonic and eudaimonic perspectives [57].

Subjective well-being stemming from the hedonic view of well-being highlights that individuals pursue pleasure and avoid pain, thus determined by emotions related to happiness, satisfaction, and interest in life [58,59,60]. In contrast, eudaimonic well-being, which originated from the tradition of Aristotle’s spirit, believes that realizing one’s potential is the most vital component of well-being. Based on this idea, the concept of psychological well-being, a state of personally maintaining an optimal function, consists of six positive functional elements—self-acceptance, the purpose of life, autonomy, positive relationships with others, control over the environment, and personal growth—was proposed [57]. Additionally, Keyes [61] proposed the concept of social well-being, noting that maintaining socially optimal functions and resuming social relationships are paramount to individuals’ well-being. Social well-being is a subjective evaluation of the functions of an individual’s society, consisting of elements such as social cohesion, acceptance, self-realization, contribution, and integration [61,62].

Keyes considers well-being in terms of mental health, defining it as a state of pursuing happiness without a mental disorder, not simply a mental problem [59]. From such a standpoint, Keyes created the concept of mental well-being by combining all three well-being concepts and theories [59,63]. Consequently, mental well-being theory has become one of the most widely used and validated frameworks in psychology.

The impact of social interaction on well-being has been well-documented in various studies [64,65,66]. These studies have highlighted the significant influence of interactions not only with close friends and family but also with acquaintances, such as weak ties, on well-being [67]. This underscores that online interactions in cyberspace can influence well-being [68], and interactions with fellow fans within an online fan community may also have a meaningful impact on well-being. Furthermore, parasocial relationships can also contribute to contribute to well-being [69]. The presence of artists, or even the mere act of thinking about them, can provide a sense of social support and refuge [70]. These insights suggest that parasocial relationships with celebrities can be vital elements in promoting well-being. Thus, we suppose that the intensity of online interaction with other fans and parasocial relationships with celebrities will be positively and significantly related to the mental well-being of fans.

**H3.**  *Intensity of online interaction with other fans will be positively related to mental well-being.*

**H4.**  *Parasocial relationships with celebrities will be positively related to mental well-being.*

### 2.4. Sense of Virtual Community (SOVC)

The field of community psychology regards the concepts of a sense of community (SOC) as well as well-being as fundamental pillars, serving as central elements for theory, research, and practical intervention [71]. It is seen that the sense of community and well-being mutually complement each other, emphasizing the equal significance of both individual well-being and the relationships individuals have within their communities [55]. The term SOC is defined as the feeling of being a part of an easily accessible, mutually supportive network of relationships [72]. SOC is a core and unique facet of community psychology, regarded as the result of communication, cooperation, and deliberation among people with similar commitments and goals in the community [73]. 

Several researchers have extended the concept of SOC into the realm of online platforms, introducing the term SOVC to explore individuals’ sense of belonging, identity, attachment, and membership within online communities that primarily engage in electronic communication [74,75]. SOVC can be defined as the community members’ feelings of membership, identification, belonging, attachment, and support in online communities [75,76]. Hence, SOVC is considered a significant constituent when building a community [77], without which the online community would be merely a virtual settlement [78].

SOVC is not a single concept but a mixed one with multiple characteristics. A basic subdimension of SOVC includes membership (the feelings of belonging), influence (the belief that members can trust and influence each other), shared emotional connections (the feelings that members receive support and form emotional bonds with each other), and fulfillment of needs (the rewards that members can get from their community membership) [30,75,76]. 

The dynamic interaction among online community users plays a significant role in building a member’s emotional attachment to the online community and solidifying the community itself [21,77]. The more frequently individuals socially interact, the stronger the ties they form [79,80]. In particular, a sense of community is considered the result of an interaction in which people with similar interests and goals gather [73]. Yang and Shim [30] concentrate on the relationship between the quality and depth of interactive communication in fan communities and SOVC, discovering that active online interaction on Weibo positively influences the forming of a cohesive fan community. Thus, if a fan’s intensity of interaction grows, a feeling of bonding or emotional attachment to the affiliated virtual community and its members may also increase [14,16,81].

**H5.**  *The intensity of online interaction within the fan community platform will positively affect the sense of virtual community (SOVC).*

In addition, as fans are given more options to interact with celebrities, they feel more emotionally connected to the celebrity and are rewarded as a fan community member [82]. A study [83] discovered that internet technologies encouraged cyclist fans to experience various dimensions of interactions and build passionate feelings for both the celebrity and the fan community. Another study [40] found that parasocial relationships formed by watching a Netflix show and repeatedly commenting on influencers’ videos facilitated feelings of unity and belonging among individuals during social isolation in the current pandemic era. Based on previous studies, this study assumes that a parasocial relationship with celebrities on fan community platforms will establish SOVC.

**H6.**  *Parasocial relationships on the fan community platform will positively affect a sense of virtual community (SOVC).*

**Figure 1:**
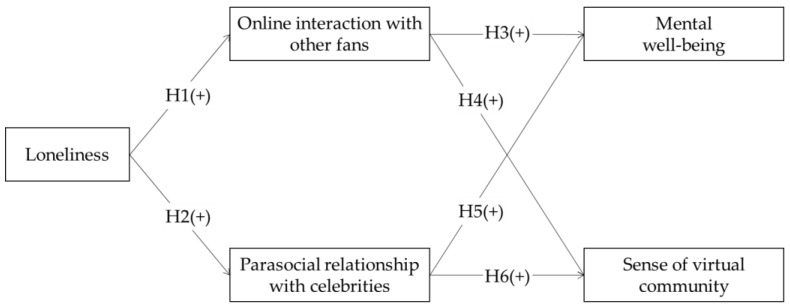
Research model.

The overall research model is described in Figure 1.

Furthermore, in fan community platforms, paid subscription groups exhibit distinct characteristics when compared to their free-access counterparts. Paid subscribers often enjoy increased access to artist interactions, exclusive content, and early access to events and products. They also have greater opportunities for intimate engagement with artists through live chats, Q&A sessions, and fan meetings. This typically includes access to exclusive content like behind-the-scenes videos, unreleased music, and videos. Furthermore, paid subscribers actively engage in community activities such as fan polls, forums, and fan clubs, fostering interactions with fellow fans. Taking these differences into account, we propose Research Question 1, which aims to investigate whether there are discernible differences in research outcomes between the paid subscription group and the free access group.

**RQ1.**  *How do the results for the hypotheses differ between paid subscribers and free users in the fan community platform?*

## 3. Materials and Methods

### 3.1. Sample and Data Collection

This study selected a fan community platform, Weverse, as the subject of analysis. Weverse is a global fan community platform developed by Weverse Company, a subsidiary of HYBE Entertainment, which is a noteworthy player in the music industry. It is a home to mega-hit groups like BTS and Blackpink. The music industry stands out as a prominent domain where fan communities are especially pronounced. Given the paramount significance of music fandom, we deemed it appropriate to focus our research on a fan community platform that specifically caters to music enthusiasts. 

Weverse stands out as an integrated platform combining social media, an official fan community, and a merchandise shop, providing an ideal environment for artist-fan interactions. While other fan community platforms offer communication functions with artists and support fan interaction services, they often encompass diverse categories such as TV programs and sports stars, making it challenging to generalize findings specific to music artists. In contrast, Weverse exclusively caters to music artists, which aligns perfectly with our research objectives. Notably, it empowers fan-to-fan and fan-to-artist communication functions through cutting-edge information technologies. Moreover, Weverse’s acquisition of the live streaming service, V Live, in 2022 further enhances its capabilities and appeal within the platform [82]. This strategic move positions Weverse as an excellent platform to observe and analyze the behaviors and sentiments of fans in the evolving landscape of fan community platforms. 

The targeted participants are the members of the ARMY, an official fan community for the South Korean musician group, BTS. This is a seven-member group that has quickly gained popularity overseas since its debut in 2013. BTS’s fandom group, ARMY, is also a global, large, and diverse community. ARMY’s dedication and support have made BTS successful and popular worldwide [26]. Additionally, ARMY has the biggest number of fan subscribers in Weverse. As a subsidiary company of HYBE Entertainment, Weverse has grown with BTS since its first launch. Accordingly, BTS Weverse now serves as an official fan club, having replaced the position of the portal sites-based official online fan community. ARMY is considered appropriate as a sample for this study for the above reasons.

The study employed a multi-faceted approach to recruit a diverse sample of global ARMY fans for participation. The primary methods included the utilization of various social media platforms, such as Twitter, Instagram, and Facebook, as well as the official Weverse platform, which is a central hub for ARMY fan engagement. This approach ensured that the survey reached ARMY fans across different online spaces. In addition to online promotion, the study sought to maximize participation by collaborating with educational institutions. After obtaining the necessary permissions, the survey was promoted during university lectures at Korea University, Pukyong National University, Gachon University, and Kyungpook National University. This academic outreach aimed to engage ARMY fans within the academic community who may not have been reached through online platforms. To initiate survey participation, invitations were distributed through email communications and by sharing SNS posts that contained links to the questionnaire. These invitations were designed to be easily shareable among the ARMY community to encourage participation.

The snowball sampling procedure cannot claim its generalizability due to the lack of representative samples within the research group [84]. However, it is considered ideal when conducting research at a low cost, in a wide geographic area, or in a group thus far not studied [85]. Also, several other studies implemented the snowball sampling method to collect global fan data, including BTS fans [86], metalhead fans [85], and soap opera fans [25]. In seeking to minimize social desirability bias, we asked participants to answer all of the survey questions anonymously [87].

Overall, among 346 responses received from 7 April to 22 May 2022, 202 completed surveys were accumulated from a web-based survey using Qualtrics online software. It was revealed that 112 respondents were left in the middle of the survey, and a screener question filtered 32. Most survey participants were female (86.1%) and under 30 (86.6%). ARMY from 35 countries, including Korea, participated in the survey. Specifically, the countries involved include the US, India, Canada, Brazil, the Philippines, Germany, and Bulgaria. ARMY members mainly joined a USD 22 subscription plan (48%), and about a third did not join the club (36.6%). Most fans seem to visit Weverse 3–5 times a week (68.3%). Additionally, it turns out that Weverse is the most-used social media platform among ARMY fans for interacting and communicating with BTS. In particular, Weverse has been confirmed to be the space where fan-celebrity interactions occur. The overall participant demographics are reflected in Table 3.

### 3.2. Measurements

All constructs were measured with multiple items developed and tested in the existing literature. The measurement items were borrowed and adapted to fit the Weverse context. The items were anchored on a 5-point Likert scale, ranging from “1” (strongly disagree) to “5” (strongly agree).

The measurement items for the intensity of online interactions with other fans were adapted from the previous literature [16,17,36] and modified to the Weverse context. We used scales for the parasocial relationship with celebrities from Rubin and Perse [35] and Kim et al. [17], which define the relationship as a friendship, and Chung and Cho [36], who considered it as understanding. SOVC constructs were derived from McMillan and Chavis [79], Kim et al. [17], and Blanchard [75], which measured subdimensions of SOVC independently. Previous studies have utilized various assessments of SOC with diverse factor structures, including unidimensional, three-dimensional, four-dimensional, and five-dimensional models [71]. While many of the earlier SOC measures were unidimensional [71,88], there has been a growing consideration of subdimensions. However, depending on the research objectives and methodological appropriateness, researchers sometimes choose to aggregate these dimensions into a single composite indicator [89,90]. In this study, we integrated the dimensions into one SOVC scale, including membership, shared emotional connection, and fulfillment of needs, that seems to fit in the Weverse context. For mental well-being, the measurement scales created by Keyes [59,63] were considered, known as the Mental Health Continuum Short Form: MHC-SF. For the Korean version of the survey, this study used the Korean version of MHC-SF (K-MHC-SF) created and validated by Lim et al. [91]. The variables and their constituent items mentioned above are well established and validated in the previous literature. Therefore, we conducted the main survey without a separate pre-test.

### 3.3. Data Analysis

We used the software application SmartPLS 4 to test the effectiveness of our research model. The present study adopted partial least squares (PLS) as a soft modeling approach to structural equation modeling (SEM) that can verify the suitability of measurement constructs and their causal relationships expressed through hypotheses [92]. PLS–SEM is a promising method for exploratory studies that are willing to analyze causes and predictions between variables or test and develop an early stage of a theory [93]. Moreover, prior studies deployed PLS–SEM when encountering a condition with a small sample size, somewhat skewed data distribution, and complex structural models [93,94]. One commonly employed approach for estimating the minimum sample size in PLS-SEM is the “10-times rule” method, as outlined by Hair et al. [93]. This method is based on the principle that the sample size should exceed ten times the highest number of connections, either inner or outer model links, directed at any latent variable within the model [95]. Similarly, as one of the exploratory studies, we applied the PLS–SEM approach to examine small and non-normal datasets and developed a new structural model created to explain fandom behavior and attitudes in a newly-made global fandom app.

## 4. Results

### 4.1. Measurement Model

We used a reflective measurement model in this study, given that the indicators are highly correlated and interchangeable. For this purpose, we thoroughly investigated the reliability and validity of the measurement constructs [93,96]. In PLS-SEM, the measurement model is evaluated using reliability, convergent validity, and discriminant validity as assessment criteria. Internal consistency reliability of the items was assessed with Cronbach’s alphas and composite reliabilities (CR) [94]. The current study first checked indicator reliability to ascertain that the loading of an indicator’s values is greater than the acceptable level of 0.7 [93]. All indicator loadings were larger than 0.7. Table 4 shows that all constructs were higher than the minimum value of 0.7 for Cronbach alphas (0.747–0.924) and composite reliabilities (0.855–0.941). We evaluated convergent validity using the average variance extracted (AVE), factor loading, and CR. A commonly accepted threshold for AVE is 0.5 or higher [97], while a factor loading and CR of 0.7 or higher are typically considered adequate [98]. As indicated in Table 4, all values confirmed convergent validity, with AVE values ranging between 0.649–0.851. Discriminant validity was evaluated by checking whether the square root of AVE in each construct is larger than other correlation values among the constructs (Table 5) [92]. Indicator’s loadings were also considered, where they should be higher than all of the other constructs’ cross-loading values (Table 6).

### 4.2. Structural Model

The results showed that loneliness has a considerable positive relationship with the intensity of online interaction with other fans (β = 0.267, *p* < 0.001) but no significant impact on the parasocial relationship (β = 0.037, *p* = 0.672). Thus, while H1 was supported, H2 was not.

We then examined the factors that would impact a fan’s mental well-being and SOVC in the new fandom platform: intensity of online interaction with other fans and parasocial relationship with celebrities. The research results indicated that the intensity of online interaction with other fans has a positive and robust effect on mental well-being (β = 0.321, *p* < 0.001) and SOVC (β = 0.569, *p* < 0.001). The data were consistent with H3 and H4.

This study also hypothesized that parasocial relationships with celebrities in the fan community platform would positively influence mental well-being and SOVC. The results showed that the parasocial relationship has a positive relationship with both mental well-being (β = 0.181, *p* = 0.029) and SOVC (β = 0.327, *p* < 0.001). Thus, the data were consistent with H5 and H6. Table 7 summarizes the relevant results, including hypotheses, coefficients, and t-values.

### 4.3. Differences between Paid Subscribers and Free Users

Research question 1 aimed to explore whether the results for the hypotheses vary across paid subscribers and free users. The findings are presented in Table 8 and Table 9.

In the paid subscriber group, except for the influence of parasocial relationships on mental well-being, the results were consistent with those in the overall group. For loneliness, a significant influence was identified on online interaction (β = 0.349, *p* < 0.001), while it did not significantly enhance parasocial relationships with celebrities (β = 0.091, *p* = 0.377). In the paid subscriber group, it was observed that online interaction with other fans had a significant impact on both mental well-being (β = 0.491, *p* < 0.001) and SOVC (β = 0.608, *p* < 0.001). However, parasocial relationships with celebrities were found to have a significant impact only on SOVC (β = 0.301, *p* < 0.001), not on mental well-being (β = 0.102, *p* = 0.370).

In the free user group, it was revealed that loneliness did not have a significant impact on either online interaction with fans (β= 0.148, *p* = 0.279) or parasocial relationships with celebrities (β = −0.048, *p* = 0.780). Furthermore, online interaction was found to not significantly affect mental well-being (β = 0.086, *p* = 0.581) but rather only enhanced SOVC (β = 0.520, *p* < 0.001). However, parasocial relationships were observed to significantly improve both mental well-being (β = 0.312, *p* = 0.011) and SOVC (β = 0.359, *p* = 0.001).

## 5. Discussion and Conclusions

As a fan community platform created for artists and fans, Weverse presents a new paradigm of social media platforms in a global market and is attracting worldwide attention regarding their usefulness and effectiveness. In particular, the growth of Weverse has been possible with the support of K-pop artists and fans as well as entertainment agencies that hoped to make a profit within the platform. Notably, as the role and scale of offline communities have been shrunk due to COVID-19, the influence of taste-based online communities like Weverse has been vastly enhanced, making the phenomenon worth examining. To this end, this study focused on the case of Weverse and the BTS fandom, ARMY, which show the most remarkable presence within the medium. We investigated the impact of loneliness in the context of COVID-19 on online interactions with other fans and parasocial relationships with celebrities on the Weverse platform. Additionally, we examined how these interactions influence the two core pillars of community psychology: well-being and SOVC.

The results revealed that loneliness significantly increased online interaction with other fans but had no significant impact on parasocial relationships with celebrities. One possible explanation for the lack of a significant relationship between loneliness and parasocial interaction is that fan community platforms like Weverse may not be as effective as other mediated communication channels in fulfilling interpersonal needs [99]. While these platforms facilitate interactions among fans and with celebrities, the quality of communication, often involving translations and one-to-many interactions rather than one-on-one private chats, may not always align with the context or depth of the conversations fans seek. Both interaction with other fans and parasocial relationships were found to significantly increase well-being and SOVC. Fan interactions centered around shared interests and passions, providing fans with opportunities to express themselves, share their enthusiasm, and receive positive reinforcement from like-minded individuals. Fans can also form a supportive network that offers emotional encouragement, understanding, and a sense of acceptance, all contributing to improved mental well-being and SOVC.

However, intriguingly, when comparing the paid subscription group and the free user group on Weverse, we found different outcomes. In the paid subscriber group, parasocial relationships with celebrities did not significantly impact fans’ mental well-being. The perception that these relationships were transactional, and the awareness that they could be terminated at any time by canceling the subscription, might have contributed to this result, deepening the superficial nature of these connections. The remaining relationships within the paid subscriber group were consistent with the overall group. 

On the other hand, in the free user group, loneliness did not significantly impact either online interaction with other fans or parasocial relationships with celebrities. Paid subscribers may exhibit a higher level of commitment to the platform and its members due to their financial investment, which could result in a stronger incentive to interact. Conversely, free users may engage primarily out of pure interest or for entertainment purposes. Therefore, loneliness might play a less prominent role as a motivator for this group, diminishing its impact on their level of interaction. Furthermore, in the free user group, online interaction was unable to significantly impact well-being, whereas in the paid subscriber group, parasocial relationships also did not significantly influence well-being. In the free user group, it is possible that the quality of interactions, the depth of relationships formed, and the extent of emotional support exchanged were lower compared to the paid subscriber group. This could imply that some free users might have experienced interactions that were more superficial or less supportive, leading to a reduced impact on their well-being.

This study establishes a foundation for comprehending the complex interplay of loneliness, fan interactions, and well-being within the dynamic realm of fan communities and global pandemics, offering both theoretical and practical insights. From a theoretical perspective, this study takes the first step in extending the existing literature on the online fan community to the fan community platform context, a new mobile app and web platform paradigm specializing in providing artist-to-fan communications. Specifically, this study employs the concepts of online interaction, parasocial relationships, mental well-being, and SOVC to illustrate the phenomenon in a virtual fan community and incorporate them into one research model. In this regard, it improves our academic understanding of fandom by defining and dealing with the characteristics of the fandom platform precisely, focusing on the service features. Consequently, applying SOVC in other contexts of relational communities seems crucial to broadening the understanding of SOVC and its positive effects. This study was an attempt to expand the literature on SOVC with empirical evidence.

Although well-being from the mental health approach can be a vital consequence of the users’ media use, it has rarely been investigated in fan studies or media industry studies. Some research using the construct of well-being has only focused on specific dimensions, including emotional well-being, such as pleasure and satisfaction, psychological well-being, such as self-fulfillment, or social well-being. It was usually the health science disciplines that used the mental well-being scale. Taking the health crisis on an individual’s ill-being during the current pandemic, mental well-being, which focuses on the pursuit of happiness in a state without a mental problem, could become the most appropriate facet to understanding people’s behaviors and attitudes toward emerging media services.

From a managerial perspective, this study offers valuable insights and practical implications for fan community platform operators and entertainment agencies. In the evolving landscape of fan communities, platforms like Weverse have transformed into influential entities within the media industry, impacting artists, content, and marketing strategies. The abundance of study results underscores the growing need for more sophisticated and diverse communication functions within these platforms.

The study’s identification of potential transactional aspects within paid subscription models emphasizes the importance for fan community platform operators to recognize how users perceive these relationships as commodities that can be terminated, potentially affecting the depth and authenticity of their connections. To address this challenge, platforms can explore strategies that make these interactions more genuine and enduring while maintaining a balance between offering exclusive content and preserving authentic connections.

Moreover, the study highlights the varied impact of loneliness on user engagement, particularly between paid subscribers and free users. This underscores the necessity for platform operators to understand and cater to the diverse motivations of these user groups. Platforms should consider tailoring their features and offerings to align with the multifaceted motivations exhibited by these users.

Additionally, fan community platform operators should prioritize efforts to enhance the depth and quality of interactions within free user groups. By doing so, they can foster more meaningful conversations, provide emotional support, and cultivate a stronger sense of community, regardless of subscription status. The study also anticipates that the parasocial relationship between fans and celebrities will continue to intensify, especially with the integration of real-time live-streaming functions led by celebrities. Furthermore, the attachment fans have for celebrities can be enhanced through direct and dynamic interactions facilitated by future ICT technologies, such as the Metaverse.

Even though the current study suggests some meaningful implications, it also entails several limitations that leave room for further studies. First, the external validity of our research samples should be carefully reviewed, notwithstanding their sufficient size (N = 202). We attempted to accumulate global fan data, but the survey could only encompass fans who can read and understand English or Korean. Moreover, it should be noted that ARMY is a unique fandom with outstanding loyalty and pride to its artist, BTS, and the fandom itself [100]. Hence, their attitudes and behaviors toward this topic may not be the best representative responses for all of the fandom population. Therefore, future studies are likely to be conducted to discover whether similar patterns are found in other parts of the world, which can translate the same survey questionnaires into other languages. Moreover, additional studies on other celebrities’ fandom, such as K-pop girl group fandom, as well as contexts featuring diverse and larger samples and settings, seem necessary to confirm the generalizability of this study. Next, we focused on the case of Weverse without discussing similar fandom services, such as LYSN & Bubble or Universe. The lack of general applicability of the findings may limit the value of our results. 

Accordingly, as other fan community platforms differ in details and core values, future studies could disclose fans’ perceptions, attitudes, and behaviors on these media services. In addition, while the current study discovered significant influencers that could explain fans’ mental well-being and SOVC, other possible factors can also affect these variables. Thus, future research may be conducted beyond interaction-related factors. It may include variables of more diverse dimensions, such as trust or the quality of content and information. Finally, investigating the core features and analyzing consumers’ willingness to pay for fan community platforms may lead to valuable and intriguing findings in future research.

## Figures and Tables

**Table 1:** Example of K-pop celebrity’s fandom.

Fandom	ARMY	Blinks	NCTzen
Genre	K-pop	K-pop	K-pop
Artist	BTS	Black Pink	NCT
Year Established	2013	2016	2017
Management Agency	HYBE	YG	SM
Social Media	Twitter, YouTube, Instagram, Weverse	Twitter, YouTube, Instagram, Weverse	Twitter, YouTube, Instagram, LYSN
Official fan community	Weverse	Weverse	LYSN/Bubble
Merchandise shop	Weverse shop	Weverse shop	SM Town and Store
Members	14.6 million (Wever)	2.8 million (Wever)	Unknown

**Table 2:** Example of fan community platforms.

Platform	Weverse	LYSN & Bubble	Universe
Company	HYBE	Dear U (SM)	NC Soft
Company Type	Entertainment company	Entertainment company	Video game developer
MAU *	6.8 million	Differ by each app	4.4 million
Number of Artists	43	249	32
Features	Official e-commerce (Weverse shop), Acquisition of V Live **	Direct celebrity to fan message	Original content (Universe Original), Digital currency (Clap)
Communication with artists	Artists’ posts, Comments on artists’ posts		
_Free users	Story function	Change artist profile	Change artist profile, Separate posts by member
_Subscribed users	Exclusive member content, merchandise, early-bird tickets	Video fan sign Artists’ handwritten letter	Artists’ private message (one-to-many), AI voice message
Communication with other fans	Fans’ posts		
	Subscribe to other fans’ accounts	Open chat	Subscribe to other fans’ accounts

* MAU: Monthly Active Users. ** V Live: a live-streaming platform for K-pop artists.

**Table 3:** Participant demographics.

Measures		Frequency	Percent
Gender	Male	28	13.9
	Female	174	86.1
Age	18–24	120	59.4
	25–30	55	27.2
	31–40	13	6.4
	Over 40	14	6.9
Nationality	Korean	80	39.6
	Not Korean	122	60.4
Frequency of Weverse visit	Everyday	67	33.2
	3–5 times a week	71	35.1
	1–2 times a week	58	28.7
	I do not visit even once a month	6	3
Membership subscription	ARMY Membership (22.00 USD)	97	48
	ARMY Membership (160.00 USD)	31	15.3
	No subscription	74	36.6
Social media used the most for interaction with ARMY	Twitter	68	33.66
	YouTube	18	8.91
	Weverse	77	38.12
	Instagram	33	16.34
	V Live	6	2.97
Social media used the most for interaction with BTS	Twitter	15	7.43
	YouTube	16	7.92
	Weverse	108	53.47
	Instagram	37	18.32
	V Live	26	12.87
Total		202	100.0

**Table 4:** Reliability and validity results *.

Construct		Factor Loading	AVE	CR	Cronbach’s α
Loneliness	LO1	0.926	0.851	0.920	0.825
	LO2	0.919			
Online interaction with other fans	OI1	0.864	0.725	0.941	0.924
	OI2	0.816			
	OI3	0.788			
	OI4	0.873			
	OI5	0.850			
	OI6	0.915			
Parasocial relationship with celebrities	PR1	0.818	0.673	0.860	0.759
	PR2	0.807			
	PR3	0.836			
Mental well-being	MW1	0.779	0.649	0.902	0.866
	MW2	0.849			
	MW3	0.794			
	MW4	0.815			
	MW5	0.790			
Sense of virtual community	SOVC1	0.823	0.663	0.855	0.747
	SOVC2	0.841			
	SOVC3	0.778			

* AVE = average variance extracted; CR = composite reliability.

**Table 5:** Correlations of the constructs and square root of AVE *.

	LO	OI	PR	MW	SOVC
LO	(0.922)				
OI	0.268	(0.851)			
PR	0.035	0.243	(0.820)		
MW	0.094	0.360	0.254	(0.806)	
SOVC	0.103	0.643	0.465	0.255	(0.814)

* The numbers in parentheses are the square root of AVE. The numbers not in parentheses are correlation. LO = loneliness, OI = online interaction with other fans, PR = parasocial relationship with celebrities, MW = mental well-being, SOVC = sense of virtual community.

**Table 6:** Cross-loading table for the reflective constructs *.

	LO	OI	PR	MW	SOVC
LO1	0.926	0.248	0.068	0.096	0.082
LO2	0.919	0.246	−0.005	0.076	0.108
OI1	0.232	0.864	0.228	0.333	0.539
OI2	0.193	0.816	0.131	0.248	0.509
OI3	0.191	0.788	0.227	0.239	0.550
OI4	0.237	0.873	0.153	0.304	0.545
OI5	0.265	0.850	0.221	0.328	0.525
OI6	0.244	0.915	0.269	0.374	0.614
PR1	0.056	0.268	0.818	0.205	0.405
PR2	0.079	0.117	0.807	0.170	0.306
PR3	−0.036	0.195	0.836	0.240	0.417
MW1	0.093	0.222	0.224	0.779	0.146
MW2	0.114	0.333	0.149	0.849	0.213
MW3	−0.012	0.182	0.221	0.794	0.200
MW4	0.034	0.295	0.205	0.815	0.122
MW5	0.119	0.367	0.227	0.790	0.315
SOVC1	0.116	0.653	0.284	0.284	0.823
SOVC2	0.050	0.488	0.449	0.175	0.841
SOVC3	0.082	0.406	0.420	0.151	0.778

* Abbreviations are same as in Table 5.

**Table 7:** Results of hypothesis testing ^1^.

Hypothesis	Path	*β*	*t*	Result
H1	LO → OI	0.267 ***	3.905	Supported
H2	LO → PR	0.037	0.424	Rejected
H3	OI → MW	0.321 ***	4.496	Supported
H4	OI → SOVC	0.569 ***	10.781	Supported
H5	PR → MW	0.181 *	2.182	Supported
H6	PR → SOVC	0.327 ***	5.429	Supported

^1^ Abbreviations are same as in Table 5. * *p* < 0.05, *** *p* < 0.001.

**Table 8:** Results of hypothesis testing for paid subscribers ^1^.

Hypothesis	Path	*Β*	*t*	Result
H1	LO → OI	0.349 ***	4.413	Supported
H2	LO → PR	0.091	0.884	Rejected
H3	OI → MW	0.491 ***	6.322	Supported
H4	OI → SOVC	0.608 ***	9.974	Supported
H5	PR → MW	0.102	0.897	Rejected
H6	PR → SOVC	0.301 ***	4.056	Supported

^1^ Abbreviations are same as in Table 5. *** *p* < 0.001.

**Table 9:** Results of hypothesis testing for free users ^1^.

Hypothesis	Path	*β*	*t*	Result
H1	LO → OI	0.148	1.083	Rejected
H2	LO → PR	−0.048	0.280	Rejected
H3	OI → MW	0.086	0.552	Rejected
H4	OI → SOVC	0.520 ***	5.490	Supported
H5	PR → MW	0.312 *	2.533	Supported
H6	PR → SOVC	0.359 ***	3.488	Supported

^1^ Abbreviations are same as in Table 5. * *p* < 0.05, *** *p* < 0.001.

## Data Availability

The data are available from the corresponding author upon reasonable request.
